# Atypical Impact of Action Effect Delay on Motor Performance in Autism

**DOI:** 10.1007/s10803-023-06227-9

**Published:** 2024-02-05

**Authors:** Noam Karsh, Marissa Hartston, Bat-Sheva Hadad

**Affiliations:** 1 Department of Psychology, Tel-Hai Academic College, Upper Galilee, Israel; 2 Special Population Advance Research and Clinical Center (SPARC), University of Haifa, Haifa, Israel; 3 Department of Special Education, Edmond J. Safra Brain Research Center, University of Haifa, Haifa, Israel

**Keywords:** Autism, Control, Sensorimotor, Predictions, Agency, Motor performance

## Abstract

Atypical sensory perception and motor impairments are primary features of autism spectrum disorder (ASD) that indicate atypical development and predict social and non-social challenges. However, their link is poorly understood. Sensory perception is often integrated with motor processes when a sensory effect is temporally contiguous with the motor response. Such sensory-motor coupling further improves motor behavior. Previous studies indicate alterations in sensory perception of action-effect temporal contiguity in ASD, which bares the question of how it may impact motor performance. People diagnosed with ASD and typically developed (TD) participants performed a speeded reaction-time task previously established to capture the facilitating impact of action’s perceptual effect on motor response selection. The sensitivity of this mechanism to delays in the effect was measured, manipulating the action-effect temporal contiguity in a within-subject design. An immediate action effect (compared to a No-effect condition) facilitated response selection in the TD group. This facilitation effect was evident in the ASD group but did not show the typical sensitivity to the effect delay. While in the TD group, RT was shorter in the short (225ms) compared to the long (675ms) action effect delay condition, this distinguished pattern was absent in the ASD group. The findings provide supporting evidence that atypical motor performance in ASD results, at least in part, from an altered sensory perception of action effect temporal contiguity. We discuss the results in light of the reduced perceptual specialization account in ASD and its potential for undermining adaptive sensorimotor processes.

Autism spectrum disorder (ASD) is a neurodevelopmental condition mainly characterized by deficits in social interactions, repetitive motor behaviors, and restricted personal interests (DSM-5; American Psychiatric Association, 2013). Alterations in sensory perception and motor impairments are primary features in ASD (for a recent comprehensive review, see Hadad & Yashar, 2022) that may underlie a large portion of symptoms in the social, behavioral, and affective domains (Van de Cruys et al., 2014; Hannant et al., 2016a; Linkenauger et al., 2012; Cook et al., 2013). Although sensory and motor impairments provide the first signs of atypical development (see also Sutera et al., 2007), there is limited research on the relation between sensory perception and motor domains of functioning in ASD, posing a central challenge in autism research (Robertson & Baron-Cohen, 2017).

As elaborated next, sensory perception is linked to motor behavior by action-effect ( e.g., a visual, auditory, or tactile sensory feedback that follows one’s action) that plays a vital role in goal-directed motor planning, response selection, and motor control (e.g., Rochat, 1998; Wolpert & Flanagan, 2001; Elsner & Hommel, 2004; Hauf et al., 2004; Eitam et al., 2013; Karsh et al., 2016). For instance, two-month-old infants can learn the contingency between their motor action and a perceptual effect (e.g., visual feedback) and use action-effect representation to generate movement to produce the perceptual effect intentionally (e.g., Watanabe & Taga, 2006). However, the vast inflow of sensory inputs constantly challenges the sensorimotor system in differentiating self-caused from externally generated sensations. Integrating a motor action with its sensory effect depends (among others) on the temporal contiguity between the action and the perceptual effect, which indicates their causal relationship (Blakemore et al., 1999b; Eitam et al., 2013; Karsh et al., 2016; Wen, 2019). Previous work suggests there are alterations in sensory perception of action-effect temporal contiguity in ASD (van Laarhoven et al., 2019; see also, Zalla & Sperduti, 2015), which bares the question of how such alterations may impact motor performance.

Self-related sensory perception is commonly demonstrated by the sensory attenuation (SA) and the intentional binding (IB) effects, also considered implicit measures of agency (Synofzik et al., 2008; Moore & Obhi, 2012). For instance, the sensory attenuation (SA) effect refers to the attenuation of self-produced sensations (e.g., compared to a passive condition, delayed or spatially unpredictable sensory effect; Blakemore et al., 1998, 1999a; Frith et al., 2000). The IB effect refers to the perceived temporal attraction of self-produced action with a temporally contiguous perceptual effect (compared to externally produced and, in certain conditions, delayed effect; Haggard et al., 2002; Ruess et al., 2018; see also, Tanaka et al., 2019). Both measures are also sensitive to non-motor cues (e.g., contextual cues), and action-effect delay may interact with other factors that may reverse its impact, especially on temporal binding (for review, see Wen, 2019). Thus, although the mechanism(s) underlying these measures is still unclear, there is a broad consensus that they reflect the perception of action and effect causal relationships.

Studies investigating the sensory attenuation and intentional binding effects in ASD showed an attenuated impact of action effect on these measures. For instance, studying the IB effect, Sperduti et al. (2014) asked participants to perform a keypress response (operant) or hear a warning cue (passive condition). A perceptual effect was presented after a variable delay (250, 450, or 650ms). Participants estimated the time lapses between their action (operant condition) or the cue (passive state) and the perceptual effect. While typically developing (TD) participants demonstrated IB effect, which appeared more substantial for 250ms compared to 650ms (visual) action-effect delay, individuals with ASD showed no IB effect for visual stimuli for all delay conditions.

Another study investigated the sensory attenuation effect of self-produced compared to externally produced sensation in TD individuals and individuals with ASD (van Laarhoven et al., 2019). In this study, participants were trained to click on a mouse button at a fixed tempo, and an auditory tone was followed by their click response (Motor-effect condition). The amplitude of the auditory N1 component was compared to an Only-effect (without a motor response) condition. TD participants demonstrated the typical attenuation of self-produced compared to externally produced tone, as indicated by a smaller amplitude of N1 component. However, no attenuation of the N1 component was detected for individuals with ASD, suggesting that sensory perception was similar for self- compared to externally generated effects.

These two studies indicate a reduced sensitivity to the agency-relevant cues (e.g., action effect and its temporal contiguity). A recent theoretical overview proposed that these findings may reflect atypical sensorimotor processing vital for linking motor action with its sensory consequence (Valori et al., 2022). It may also reflect a more general characteristic of reduced specialization of sensory perception that complies less with statistical, ecological regularities (for a review, see Hadad & Yashar, 2022). Consistent with these claims, individuals with ASD showed modulated temporal binding in a non-motor task, demonstrating an extended time window for multisensory integration in the flash-beep illusion (Foss-Feig et al., 2010).

Temporally contiguous action effect has a vital role in the development of sensorimotor representations and enhancement of motor control performance (Eitam et al., 2013; Karsh et al., 2016; Karsh et al., 2015a; Hemed et al., 2019; Tanaka et al., 2021; Hemed et al., 2022; Karsh et al., 2023). In the current study, we focused on such motor outcomes of the temporally contiguous action-effect and investigated whether atypical sensory perception of action-effect and its temporal contiguity alters motor performance in ASD (Valori et al., 2022; see also, Mosconi et al., 2015; Hannant et al., 2016b; Lidstone & Mostofsky, 2021). This line of research offers new insights into how atypical (action-related) sensory perception contributes to delays in motor development and motor performance challenges frequently seen in ASD.

## Action-Effect Temporal Contiguity and its Impact on Motor Control Performance

Recent empirical advances provide supporting evidence for the rewarding aspects of temporally continuous action-effect by demonstrating its enhancing impact on the speed, frequency, and precision of motor response selection (Eitam et al., 2013; Karsh & Eitam, 2015a; Behne et al., 2008; Kohrs et al., 2012; Hemed et al., 2019, 2022; Bakbani-Elkayam et al., 2019; Karsh et al., 2020; see also Manohar et al., 2017).

Such motor enhancements were claimed to stem from the output of an optimal control model known as the comparator model (Blakemore et al., 1998, 1999a; Frith et al., 2000), the possible mechanism underlying the ability to distinguish between effects caused by oneself and those generated externally. Specifically, according to the comparator model, a forward sensory prediction model is generated based on an efference copy of the motor command. The sensory prediction model is compared with the actual sensory feedback representation. Such a comparison operates at a pre-conceptual level and detects temporal and spatial discrepancies between the predicted and the actual sensory feedback. A minimal (e.g., temporal) discrepancy between the action and action effect indicates that the perceptual effect is an own-action effect (Blakemore et al., 1999a). However, sensory feedback that is lagged by more than ~ 300ms from the response may not be associated with the recent motor command (Shimada et al., 2009; Frith et al., 2000; Miall & Wolpert, 1996; Wen, 2019; Shimada, 2022). Therefore, this mechanism requires highly tuned sensory perception that differentiates meaningful (e.g., >~300ms) from negligible ( < ~ 300ms) action-effect delay for optimal operation.

The Control-based response selection framework (CBRS; Karsh & Eitam, 2015b) was proposed to suggest that a minimal discrepancy detected by this model is rewarding (see also, Stephens, 1934; Skinner, 1953; White, 1959; Wen et al., 2018) which further reinforces the relevant motor program (Hemed et al., 2022) and enhance stimulus-response association (Tanaka et al., 2021), contributing to the creation and development of sensorimotor representations.

These claims were supported in previous work where participants performed a speeded response time task and were required to respond to descending circles appearing sequentially on their monitor by pressing the relevant spatially congruent key on a keyboard as fast as possible (e.g., Eitam et al., 2013; Karsh et al., 2016). A visual action-effect could appear after their response (e.g., a brief white flash on the circle cue). A consistent pattern was demonstrated across experiments showing that a temporally contiguous effect facilitates the speed of response selection compared to a subtly lagged (> 300ms; Eitam et al., 2013; Karsh et al., 2016; Karsh et al., 2022), spatially unpredicted (Karsh et al., 2016) and a No-effect condition (Eitam et al., 2013; Karsh et al., 2016). Notably, the impact of temporally contiguous action-effect on the speed of motor response selection was insensitive to abstract knowledge regarding the outcome value of the response (monetary gain; Karsh et al., 2020) and environmental regularities when motor action was not involved (e.g., the probability of an effect given no-response; Delta-p; Hemed et al., 2022). Moreover, the facilitating impact emerged with minimal explicit knowledge about action-effect temporal contiguity mapping (Karsh et al., 2016) and depended on a specific combination between the stimulus and the response (Tanaka et al., 2021).

These findings support that the facilitating impact of temporally contiguous action effect stems from sensorimotor integration that operates within a limited range of action-effect delay. The findings also suggest that such facilitation effect (at least in this kind of tasks) is independent of more high-level non-motor self-causality attributional processes, success in task performance, motivation from tangible rewards, or explicit control knowledge.

## The Current Study

Sensory perception of action-effect and its temporal contiguity is vital for sensorimotor and motor performance. Thus, altered self-related sensory perception often reported in autism (e,g., Valori et al., 2022) may contribute to motor challenges in these individuals. We investigated here the impact of action-effect temporal contiguity on the speed of motor response selection for the first time in adults with ASD. We used a modified version of a task developed by Eitam et al. (2013), which captures the facilitating impact of the temporally contiguous action effect on the speed of motor response selection. Different from Eitam’s task, delays in the action-effect were manipulated between blocks in a within-subject design, allowing a more sensitive measurement of group differences in sensory perception of action-effect delay.

In this task, a target circle appeared on a game window in one of four locations and rapidly descended vertically to the game window’s bottom. Participants were asked to respond with the correct (spatially congruent) key as fast as possible. Their response could trigger an immediate perceptual effect (a brief white flash) on the target circle (an Immediate effect condition), at variable delays (225 ms, 450 ms, or 675ms), or continue to descend to the bottom of the game window without any perceptual change following the response (a No-effect condition). Previous work using this task with TD participants (Eitam et al., 2013; Karsh et al., 2016) has demonstrated significantly shorter response times (RT) in the immediate, compared to substantially delayed (> 300ms) and the No-effect conditions. In the context of this study, shorter RTs in the immediate effect condition compared to the lagged effect condition suggest that the sensory perception of action-effect temporal contiguity adheres to ecological constraints essential for the motor system to distinguish between self-generated and externally induced effects. Contrary to this expected pattern for the TD participants, those with ASD may demonstrate alterations in linking a motor response with its sensory consequence because of atypical sensory perception of action-effect temporal contiguity (Sperduti et al., 2014; van Laarhoven et al., 2019; Valori et al., 2022; Foss-Feig et al., 2010; Hadad & Yashar, 2022). Therefore, the impact of the action effect on RT in ASD would be less modulated by action-effect delay.

## Method

### Participants

In total, forty-two adults participated in the experiment: 21 adults diagnosed with high-functioning autism (3 females; Age: *M* = 27.36, *SD* = 6.23) and 21 TD adults (12 females; Age: *M* = 26.14, *SD* = 5.12). All participants reported normal or corrected-to-normal vision and had IQ within the normal range. IQ was assessed using the Test of Nonverbal Intelligence–Fourth Edition (TONI-4, an age-standardized test with a mean score of 100 and a standard deviation of 15; Brown et al., 2010; Ritter et al., 2011). No significant differences in IQ were found between the groups (see Table 1) (two participants with ASD and one TD participant did not complete the TONI-4 test due to technical reasons).

**Table 1:** Summary statistics of AQ, Toni-4, and ADOS

	AQ (SD, Range)	Toni-4 (SD, Range)	ADOS (SD, Range)
ASD (n = 20)	28.5 (8.53, 14–46)	102.88 (11.21, 89–121)	10.25 (2.4, 7–16)
TD (n = 20)	13.5 (5.81, 6–29)	106.68 (9.97, 87–128)	–

All ASD participants completed the Autism Diagnostic Observation Schedule-Generic (Lord et al., 2000) and met the ASD diagnosis on the Social and Communication total score (Table 1). In addition, all participants completed the Hebrew version of the Autism Quotient scale (Baron-Cohen et al., 2001). Data from one TD participant and one ASD participant was excluded from the analysis (see Pre-processing section for detailed description). Individuals with ASD exhibited, on average, higher scores than TD individuals on the AQ scale [*t*_38_ = 6.49, *p* < .001, CI_95_ (10, 19), see Table 1].

All participants provided written informed consent to participate in the study and were paid 50 NIS for an hour. We recruited TD participants through an advertisement at the University of Haifa and ASD individuals through a hostel providing living assistance for ASD individuals. The Institutional Ethics Committee at the University of Haifa approved the study (IRB: #046/20).

### Stimuli and Procedure

Participants sat in a soundproof room with dim lighting at an approximate distance of 50 cm from the computer monitor and were introduced to a modified version of a computerized task first developed by Eitam et al. (2013). In this task, a colored circle (visual angle = ~ 1.402°) appears in one of four possible horizontal locations at the top of the game window (9.5 cm X 10 cm) and rapidly descends vertically to the bottom of the game window. Participants were to place both their middle and index fingers of both hands on four designated response keys (‘S’, ‘D’, ‘H’ and ‘J’) on a standard PC keyboard and were instructed to ‘stamp’ the circles as they appear on the screen as fast as they could by pressing the correct spatially-coded key.

Notably, we manipulated the temporal contiguity between the appearance of the visual action-effect and the participants’ responses (the circle changed its color to white for 100ms and disappeared, experienced as a brief white flash). Specifically, the perceptual effect appeared immediately after the participant’s response. Three additional experimental conditions were used with varying delays between their response and the appearance of the effect: 225ms, 450ms, and 675ms lagged effect conditions. In addition, the No-effect condition was used as a control, in which no flash was presented following a response, and the circle continued its descending path. The ISI was 2000ms in all condition blocks and independent of RT. Participants completed each of the five effect conditions in separate blocks of trials; a Latin Square determined the order of the blocks.

**Fig. 1:**
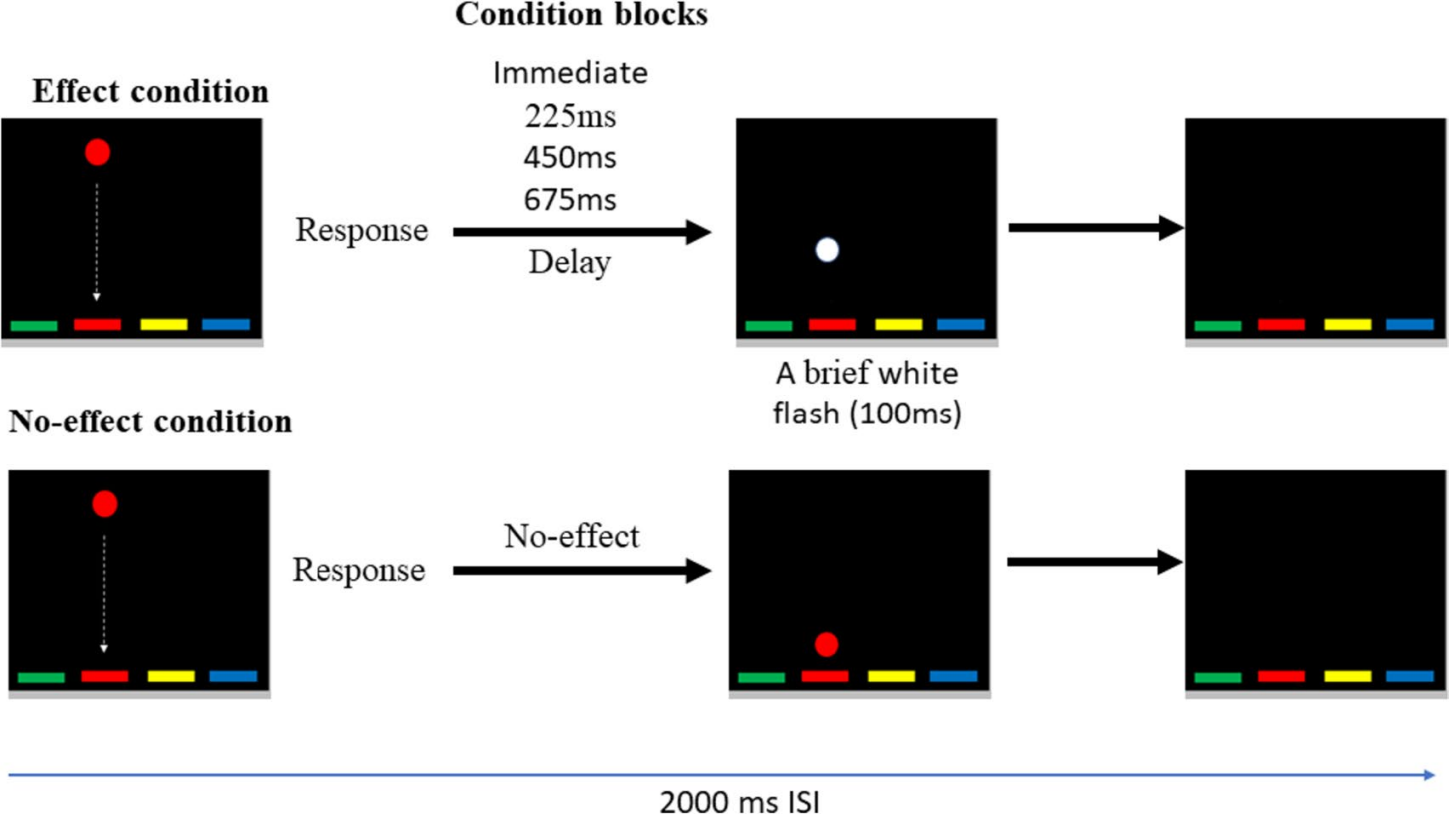
An illustration of a trial in the experimental task in each of the five condition blocks

The experimental task entailed two parts with ~ 5-minute break between the two parts. The order of the parts was counterbalanced between participants. In one part, each condition block appeared once and included 60 trials. In the other part, each of the five condition blocks included 30 trials, and this loop of five condition blocks repeated itself three times (Fig. 1). Overall, participants performed 750 trials^1^. After completing the experimental task, participants were administered the Autistic Quotient (AQ) scale (Baron-Cohen et al., 2001) and the IQ test (TONI-4; Brown et al., 2010; Ritter et al., 2011).

### Data Pre-processing

Data from one TD participant who scored 30 on the AQ scale was removed from the analysis. Similar to previous studies using this task (e.g., Karsh et al., 2020), trials with lower than 85% hit rates were removed (~ 4% of the total trials; calculated for each part of the experiment separately). There was no significant difference between ASD (*M* = 0.95, *SD* = 0.03) and TD (*M* = 0.95, *SD* = 0.02) participants in the remaining hit rates (*t*_38_ < 1). Incorrect responses (~ 4.5%), responses that were either above 700 ms or below 200 ms (~ 13%), and responses that deviated from their condition’s mean RT by at least two standard deviations from each group (~ 2%), were removed from further analysis.

## Results

Our primary dependent variable was participants’ mean response times (RT), measured in milliseconds from the circle’s appearance to the participant’s response (using Psychopy; Peirce et al., 2019). A two-way mixed model ANOVA was carried out on RTs, with Effect (Immediate, 225ms, 450ms, 675ms, and No-effect) as a within-subject factor and Group (ASD vs. TD) as a between-subject factor. The analysis demonstrated a significant difference between groups [F_(1,152)=_24.22, *p* < .001, η^2^_p_ = 0.38], demonstrating overall slower RTs in the ASD compared to the TD group (see Table 2 for descriptive statistics). In addition, replicating previous studies, a main effect of Effect was observed [F_(4,152)=_13.59, *p* < .001, η^2^_p_ = 0.26].

**Table 2:** Mean and SD of RT (ms) in the effect conditions

	TD	ASD	*Overall*
Effect condition	M (SD)	M (SD)	M (SD)
Immediate	515 (27)	570 (37)	543 (42)
225ms	522 (25)	576 (38)	549 (42)
450ms	529 (30)	576 (36)	553 (40)
675ms	535 (27)	576 (36)	556 (38)
No-effect	537 (24)	581 (36)	559 (37)
*Overall*	528 (27)	576 (36)	

**Fig. 2:**
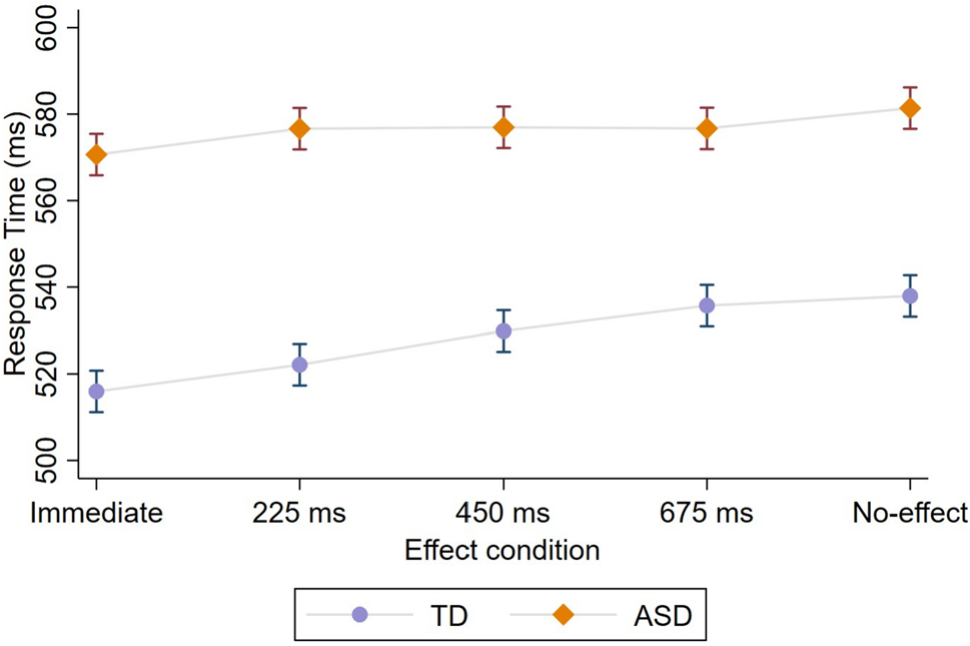
The impact of action-effect temporal contiguity on response times in the two groups. Error bars depict 95% confidence intervals

Importantly, consistent with our predictions, a significant interaction was observed between Effect and Group [F_(4,152)_ = 3.35, *p* = .011, η^2^_p_ = 0.08; see Fig. 2]. To further understand the nature of the interaction effect, we examined the difference between TD and ASD groups within each Effect condition. First, we used the No-effect as a base-level condition to test whether the groups differ in the mere facilitating impact of the action-effect. As can be seen in Table 3, both ASD and TD participants demonstrated the facilitation effect in the Immediate condition. After Bonferroni adjustment for multiple comparisons, no significant differences between groups were observed in each of the four comparisons.

**Table 3:** Group differences in the action-effect delays with the No-effect condition as baseline

	vs. No-effect	Contrast (Std. Err. = 4.85)	*P*	95% CI.	BF_10_
			(Bonferroni corrected)		
Group (TD vs. ASD)	Immediate	11	0.087	−1, 23	1.96
	225ms	11	0.095	−1, 23	0.96
	450ms	3	1	−8, 15	0.53
	675ms	-2	1	−14, 9	0.36

The contrasts depict double differences: for example, contrast 11 indicates that the difference between the Immediate and the No-effect condition is larger in the TD compared to the ASD group. We calculated Bayesian independent samples t-test on the difference between the relevant conditions between the two groups using the default Cauchy prior (= 0.707) and reported the non-directional Bayes Factor (BF)

Second, to test whether the groups differ in their sensitivity to different levels of action-effect delay, we used the (maximal) 675ms delay condition as the baseline level. As can be seen in Table 4, ASD and TD groups showed differential sensitivity to action-effect delay. Specifically, in line with previous work (Eitam et al., 2013; Karsh et al., 2016), after Bonferroni correction, RT in the TD group was shorter in the immediate [Contrast = 19.75, S.E.=3.95; *p* < .001, CI_95%_(10, 28)] and the 225ms [Contrast = 13.59, S.E.=4.29; *p* = .006, CI_95%_(3, 23)] effect condition (compared to the 675ms delay base-level condition). Unlike the TD group, RTs in the ASD group were less sensitive to different delays [Immediate vs. 675ms: Contrast = 6.04, S.E.=3.95; *p* = .27, CI_95%_(−3, 15); 225 vs. 675ms: Contrast=− 0.05, S.E.=4.29; *p* = 1, CI_95%_(−9, 10)].

**Table 4:** Group differences in the action-effect delays with 675ms delay condition as baseline

	vs. 675 ms delay	Contrast (Std. Err. =4.85)	*P*	95% CI.	BF_10_
			(Bonferroni corrected)		
Group (TD vs. ASD)	Immediate	13	0.02	1, 25	3.03
	225ms	13	0.02	1, 25	2.07
	450ms	6	0.84	-6, 18	0.81
	No-effect	2	1	-9, 14	0.36

The contrasts depict double differences: for example, contrast 13 indicates that the difference between the Immediate and the 675ms condition is larger in the TD compared to the ASD group. We calculated the Bayesian independent samples t-test on the difference between the relevant conditions between the two groups using the default Cauchy prior (= 0.707) and reported the non-directional Bayes Factor (BF)

These results suggest that an (immediate) action-effect enhances the motor performance of individuals with ASD. Still, their RTs are less modulated by different levels of action effect delays.

**Fig. 3:**
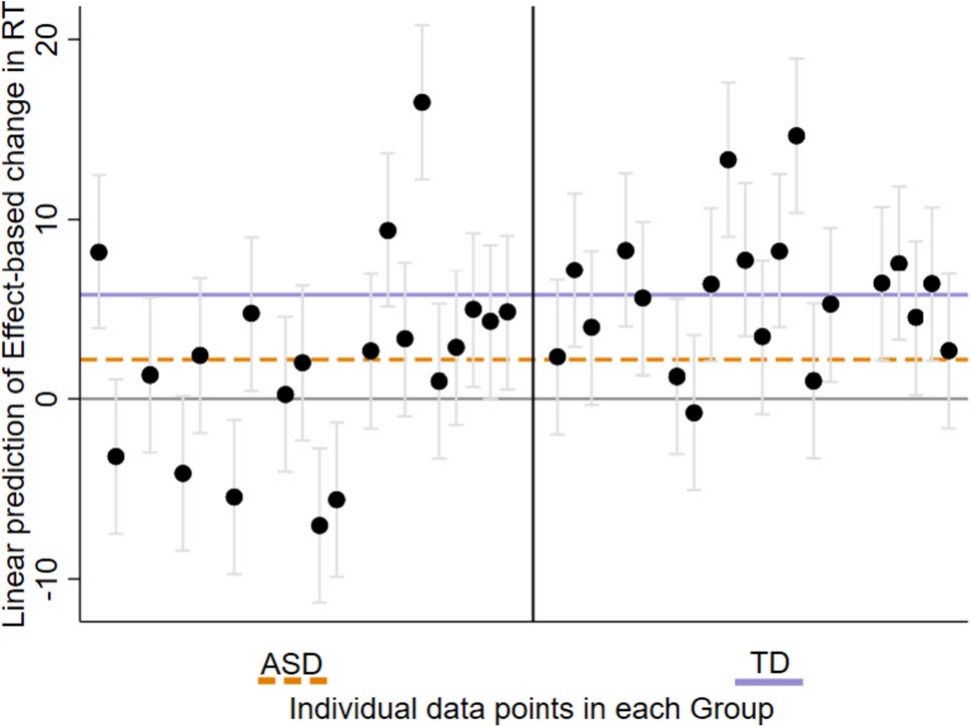
Individual data points of ASD and TD participants describing linear prediction of their averaged change in RTs as a function of an increase in action-effect delay (calculated using ‘margins’ command with ‘dydx’ option in STATA-15 software). The solid lavender horizontal line represents the averaged change in RTs as a function of increased delay for the TD group. The orange dashed horizontal line represents the averaged change in RTs as a function of increased delay for the ASD group. Data points for individuals with ASD are on the left and for the TD on the right

Finally, to estimate the predicted amount of change in RTs as a function of an increase in action-effect delay between the two groups, we used a linear mixed effect model of RTs on Effect (as a continuous predictor) and Group as fixed effects with a random intercept by the participant. The interaction between Group and Effect was significant [(Coef. = −3.6, *p* = .001, CI_95%_ (−5, −1)] suggesting that overall, the predicted amount of change in RTs as a function of an increase in action-effect delay was smaller in the ASD compared to the TD group (see also Fig. 3 for individual data).

### Autistic Quotient (AQ)

**Fig. 4:**
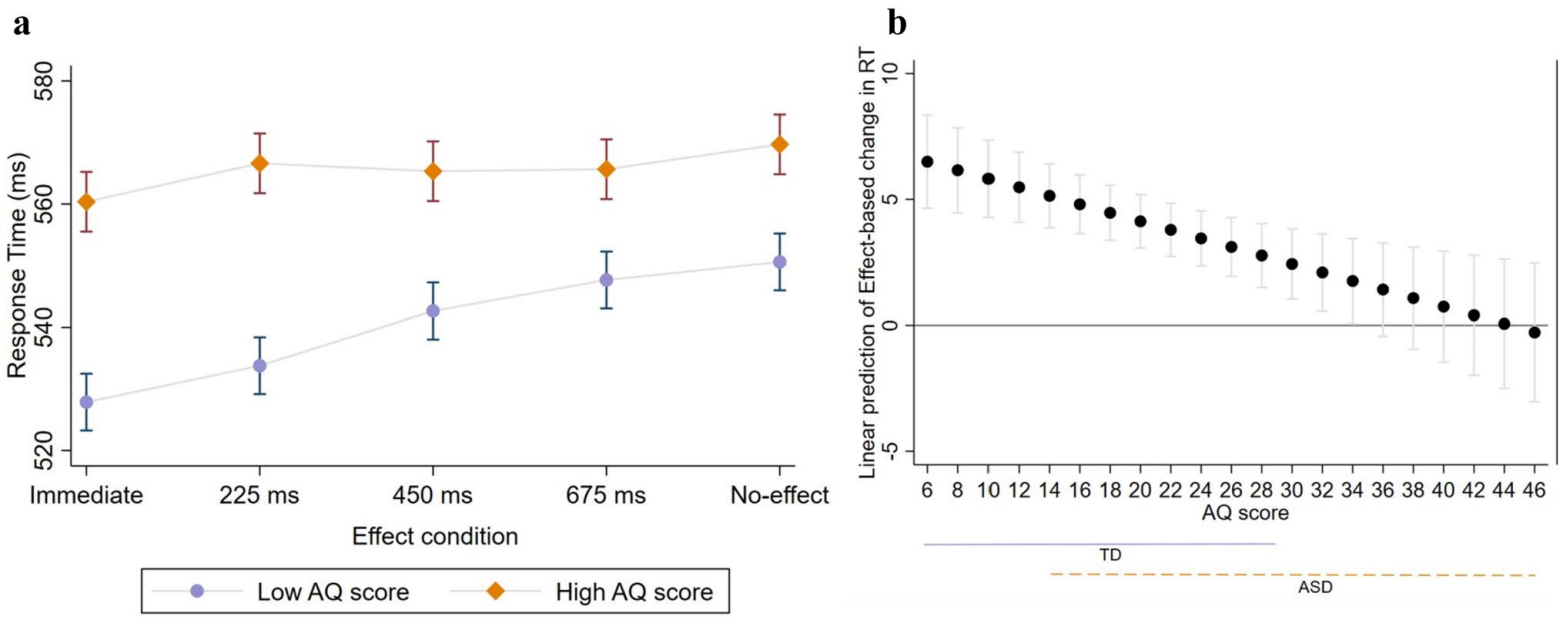
**a** The impact of action-effect and AQ on response times. Error bars depict 95% confidence interval. **b** Linear prediction of Effect-based change in RTs as a function of AQ score. Error bars represent 95% confidence interval. The solid lavender and the dashed orange horizontal lines represent the range of AQ scores in the TD and ASD groups

To explore whether Autistic Quotient (AQ) scores were associated with modulating the impact of action-effect and its temporal contiguity on RTs, we initially assigned all participants to High and Low AQ groups according to the Median score (*Med* = 18). A two-way mixed model ANOVA was carried out on RTs with Effect as a within-subject factor with five levels (Immediate, 225ms, 450ms, 675ms, and No-effect) and AQ as a between-subject factor with two levels (High vs. Low). The analysis revealed a significant difference between the High and Low AQ groups [F_(1,152)=_ 4.46, *p* = .041, η^2^_p_ = 0.1], showing slower RTs for the High (*M* = 565, *SD* = 35) compared to the Low (*M* = 540, *SD* = 40) AQ group. Interestingly, similar to the differences found between TD and ASD groups, there was a significant interaction between Effect and AQ [F_(4,152)=_4.5, *p* = .001, η^2^_p_ = 0.1]; see Fig. 4a]. To further explore whether AQ score modulated the predicted change in RTs as a function of an increase in action-effect delay, we used a linear mixed effect model of RTs on Effect and AQ score (as continuous predictors), with a random intercept by the participant. The interaction between AQ score and Effect was found significant [(Coef. = − 0.16, *p* = .001, CI_95%_ (−0.27, − 0.06)] suggesting that overall, the predicted amount of change in RTs as a function of an increase in action-effect delay level was smaller as the AQ score increased (Fig. 4b). The findings echo the observed differences between the TD and the ASD groups.

## Discussion

Atypical sensory and motor processing characterize some of the primary symptoms of ASD that may explain the etiology of their social, affective, and motor functioning. They may reflect the first signs of atypical development (e.g., Hannant et al., 2016b; Whyatt & Craig, 2013; Baranek, 1999; Linkenauger et al., 2012; Cook et al., 2013) and thus may inform early diagnosis. To better understand these alterations, we examined the motor outcome of self-related sensory perception, namely the impact of action-effect temporal contiguity on the speed of motor response selection (Eitam et al., 2013; Karsh & Eitam., 2015a; b; Karsh et al., 2016; Hemed et al., 2019; Bakbani-Elkayam et al., 2019; Karsh et al., 2020; Hemed et al., 2022). For this purpose, we used an established computerized task modified to include different levels of action-effect delays manipulated in a within-subject design. Such modification enabled a sensitive measurement of motor performance modulation by varying action-effect delay levels. The findings replicate previous work demonstrating that an immediate action-effect (compared to a No-effect condition) facilitated the speed of motor response selection for both TD and ASD participants. Although somewhat smaller, this facilitation effect was clearly evident in the ASD group, showing that motor response selection in people with ASD, as in TD, was enhanced by immediate action effects.

Following the conceptualization of the control-based response selection framework (Karsh et al., 2015b), the present findings indicate that the mechanism responsible for rewarding an (immediate) own-action effect that facilitates response-selection is intact in ASD, potentially enabling the relevant motor program’s reinforcement (Hemed et al., 2022) and the development of sensory-motor representations (Tanaka et al., 2021 or ‘event files’ Hommel, 1998). However, the attenuated impact of action-effect delay on RTs in the ASD group, may reflect an atypical sensory processing of action-effect delay that further affected motor processes. Delays in sensory feedback had altered effects on response selection in ASD. Specifically, the results in the TD group were shown to be consistent with previous studies suggesting that the facilitation of motor response selection emerges following action-effects that are lagged by less than ~ 300ms (Eitam et al., 2013; Karsh et al., 2016; e.g., the 225ms compared to the 675 ms delay condition in the current study). On the contrary, in the ASD group, RTs were almost identical in the negligible (225ms) and substantial (675 ms) action effect delay conditions.

Importantly, the forward sensory prediction model does not predict delays in the sensory feedback, which undermines sensorimotor integration (Blakemore et al., 2002; Wen, 2019). However, as discussed in our previous work (Karsh et al., 2023), inevitable delays may result from neural transmission and other internal sources that may reach up to ~ 300ms in some cases (Shimada et al., 2009; Frith et al., 2000; Miall & Wolpert, 1996; Wen, 2019; Shimada, 2022). Consistently, the facilitating impact of action-effect may typically emerge after some, yet negligible action-effect delays. Such perceptual flexibility that operates within a restricted range of action-effect delay may reflect the adaptable nature of perceptual-motor integration in promoting motor control performance (Wolpert et al., 1995, 2011; Wolpert & Flanagan, 2001). In this sense, the pattern of findings in the ASD group may indicate greater sensory sensitivity to minor action-effect delays. Thus, although the findings suggest a generally intact sensorimotor reinforcement from an immediate action-effect in ASD, in the presence of uncertainty (e.g., triggered by action-effect delay), their sensory perception is less flexible to represent action-effect delays in an ecological manner (e.g., to differentiate meaningful from negligible action-effect delays for sensorimotor processes).

Contemporary theories of sensory perception in ASD can further advance our understanding of the atypical motor pattern following delayed action-effect in the ASD group. According to the reduced perceptual specialization account in ASD, perceptual representations in ASD are considered to be too broadly tuned, operating within less specified parameters (Hadad et al., 2017; for a review, see Hadad & Yashar, 2022; Valori et al., 2022). Some aspects of the reduced specialization in ASD can be explained by the predictive coding account of ASD, which postulates a higher and inflexible weight (or precision) to the sensory input (Van de Cruys et al., 2014; Lawson et al., 2014; Palmer et al., 2017). This tendency may impair more abstract categorization of self-related sensory perception according to statistical regularities (e.g., accounting for substantial and minor action effect delays) in an ecological manner. In this sense, different delays may receive the same treatment by sensorimotor processes (e.g., the comparator model); therefore, even a minor delay (less than 300ms) may undermine sensory-motor coupling and negate the promoting impact of action effect on motor performance in the ASD group. Such an interpretation follows the findings showing shorter RTs in the 225ms condition compared to the 675ms condition in the TD but not in the ASD group. However, this conclusion awaits an independent examination of the impact of action-effect delays on developing stimulus-response-effect association in TD and ASD groups.

The present findings and interpretations can shed light on inconsistencies in the literature regarding the evaluation of action-effect predictions in ASD. For instance, previous work focusing on implicit perceptual measures of agency showed attenuated IB and SA effects in ASD, claiming an altered evaluation of agency-relevant cues in ASD (Sperduti et al., 2014; van Laarhoven et al., 2019). Another study (Finnemann et al., 2021) did not detect such attenuation, concluding that there is no general deficit in predictive processing in ASD. The findings of these studies are difficult to compare because of the very different tasks used, the different action-effect sensory modalities employed (visual, auditory, or tactile), and whether action-effect delay was manipulated. Whether these previous and present studies tap into precisely the exact mechanism is beyond the scope of the current study. Nevertheless, the present study adds to this somewhat limited literature by demonstrating reinforcement from sensorimotor predictability (Eitam et al., 2013; Karsh et al., 2016; Hemed et al., 2022) in both TD and ASD groups and highlights group differences in processing action-effect delay as a critical factor for understanding group differences in evaluating such sensorimotor predictions.

As shown for many tested functions, there was a large variability within the ASD (compared to the TD) group in the modulating impact of action-effect temporal contiguity on response times. Approximately a third of the ASD participants demonstrated an opposite trend regarding the impact of action-effect delay on RTs, a similar proportion of subjects demonstrated no noticeable impact, and the rest showed a typical effect. For the TD group, approximately 80% of the participants showed at least some facilitation effect from an immediate action-effect (see Fig. 3). Such variability within the ASD group may be considered a limitation of this study in detecting a pattern that characterizes the core ASD mechanism. However, it is also consistent with our theoretical claim for a system less constrained by ecological statistical regularities. Note that this pattern may also account for the variability in sensory symptoms within the autistic population (Happé et al., 2006; Uljarević et al., 2017), leading to apparent inconsistency between studies typically interested in groups’ mean differences. Thus, the results can set the stage for further studies on sensory phenotyping, and encourage future studies using basic perceptual and motor tasks to explore systematic variability within the autism to advance our understanding of potential ASD subtypes.

The current findings raise other potential limitations and suggestions for future research. In the present study, response times, which are typically slower and more variable (Dinstein et al., 2012) in ASD groups, were used as the primary variable indicating motor performance. Although RTs of both ASD and TD groups were shorter following an immediate effect (compared to no-effect), future work may also consider using other measures for motor performance. For example, previous work using an endpoint movement task demonstrated that temporally contiguous (compared to delayed) action-effect improved movement’s endpoint precision (Karsh et al., 2023). These kinds of tasks are less dependent on participants’ response time tendency and enable examining movement kinematics in addition to motor outcomes. Finally, it should also be considered that the attenuated impact of action-effect temporal contiguity on motor response selection in ASD may characterize a perceptual system that atypically processes temporal information (for a review, see Casassus et al., 2019) rather than a general alternation in motor-based evaluation of sensorimotor predictions. This can be examined in a future study by manipulating action-effect spatial predictability instead of temporal contiguity (Karsh et al., 2016; Exp. 2). Finally, the study procedure and requirements challenged our ability to include a larger sample of ASD participants from different levels of functioning. Future work is encouraged to use a larger sample of participants with varying levels of functioning and sensory symptoms, which may interact with action-effect delay to impact motor performance.

## Conclusions

The study integrates previous work in the perceptual and motor domains to provide novel insights on how such atypical sensory processing of an own-action effect can be related to motor performance in ASD. Specifically, the current work is the first to study the impact of action-effect and its temporal contiguity on motor performance in ASD. Like TD individuals, the motor performance of individuals diagnosed with ASD benefited from action’s immediate perceptual feedback. However, in contrast to TD individuals, the motor performance of individuals with ASD was uniformly influenced by negligible and significant action-effect delays. The findings align with the reduced perceptual specialization account in ASD, suggesting that sensory perception is less flexible to account for statistical regularities in an ecological manner. The present study supports that part of the etiology of the motor symptoms often accompanying ASD is related to sensory perception of an own-action effect.

## Footnotes

1 Note that we divided the task into two parts to explore whether the changing frequency of the condition blocks had a differential impact on ASD and TD groups performance that may have masked the influence of action-effect on RT. The pattern of findings was consistent between the two regularities and the type of experimental regularity did not interact with group to affect RT. We report separate analyses for the two parts in the Supplementary Materials.
